# Reflections from the 2021 OARSI clinical trial symposium: Considerations for understanding biomarker assessments in osteoarthritis drug development - Should future studies focus on disease activity, rather than status?

**DOI:** 10.1016/j.ocarto.2022.100262

**Published:** 2022-04-28

**Authors:** M.A. Karsdal, J. Tambiah, M.C. Hochberg, C. Ladel, A.C. Bay-Jensen, L. Arendt-Nielsen, A. Mobasheri, V.B. Kraus

**Affiliations:** a Nordic Bioscience, Herlev, Denmark; b Southern Danish University, Odense, Denmark; c Biosplice Therapeutics, San Diego, USA; d University of Maryland School of Medicine, Baltimore, MD, USA; e Independent Consultant, Darmstadt, Germany; f Center for Neuroplasticity and Pain (CNAP), SMI, Department of Health Science and Technology, Faculty of Medicine, Aalborg University, Aalborg, Denmark; g Research Unit of Medical Imaging, Physics and Technology, Faculty of Medicine, University of Oulu, Oulu, Finland; h Department of Regenerative Medicine, State Research Institute Centre for Innovative Medicine, Vilnius, Lithuania; i Department of Joint Surgery, First Affiliated Hospital of Sun Yat-sen University, Guangzhou, China; j World Health Organization Collaborating Centre for Public Health Aspects of Musculoskeletal Health and Aging, Liege, Belgium; k Duke Molecular Physiology Institute, Duke University School of Medicine, Durham, NC, USA

**Keywords:** Osteoarthritis, Drug development, Clinical trial

## Abstract

**Objective:**

Osteoarthritis (OA) is heterogeneous disease, for which drug development has proven to be challenging, both facilitated and hampered by changing guidelines. This is evident by the current lack of approved treatments, which improve joint function and delay joint failure. There is a need to bring together key stakeholders to discuss, align and enhance the processes for OA drug development to benefit patients.

**Design:**

To facilitate drug development, the Osteoarthritis Research Society International (OARSI) initiated a series of annual clinical trials symposia (CTS). The aim of these symposia was to bring together academics, translational and clinical scientists, regulators, drug developers, and patient advocacy groups to share, refine and enhance the drug development process for the benefit of patients.

**Results:**

OARSI is now considered the leading organization to facilitate open dialogue between all these stakeholders, in the intersection of understanding of the pathologies and drug development. Clearly, such a pivotal task needs an annual forum to allow stakeholders to share and discuss information, as possible solutions are joint efforts rather than a single stakeholder contribution.

**Conclusions:**

The main topic of the 2021 CTS was how to improve clinical studies to help patients through overcoming barriers to development of new disease modifying treatments for OA. One key aspect was the focus on definitions of disease activity, status and the definitions of “illness vs disease”. There is a clear medical need to couple a given disease activity with the optimal intervention for the right patient.

To develop effective disease modifying osteoarthritis (OA) drugs (DMOADs) and monitor treatment effects for the benefit of patients, we need better designed and executed clinical studies, starting from phase II clinical trials that are more mechanism based, objective, easily quantifiable, and with higher precision and accuracy. In our opinion, biomarkers that reflect disease activity are more appropriate than disease status markers for such trials. To do this, we need to understand OA from the perspective of the “disease vs illness paradigm” [1,2]. Illness represents symptoms of pain and dysfunction the patient presents with at the doctor's office, and disease is the underlying pathological driver causing the illness/pain. Recently, mechanistic pain biomarkers have shown correlation with drug effects in OA [3] and outcome after joint replacement [4], suggesting that structural and molecular drivers of pain, rather that the symptoms of pain itself, better reflect the cause of disease and disease progression.

Today, radiographs are the generally accepted measure of disease status in clinical practice, and accepted as inclusion criteria in trials, but not as a clinical endpoints or surrogate endpoint in pivotal randomized clinical trials; in general, joint images, whether radiographic or magnetic resonance images, reflect the consequences of disease activity. These seem logical as study outcomes if we want to stabilize or even reverse disease status by pharmacological interventions. However, the sensitivity of the most widely used imaging tools, such as conventional radiography, is relatively poor to detect minor changes in disease status; MRI is more sensitive [5], but we still need to link smaller cartilage changes to changes in outcome. Furthermore, there are limited tools available to measure the level of disease activity – the underlining pathologies leading to the illness, e.g., pain and inflammation, which result in the decline of joint function. These are needed to identify who to treat, when to treat, what to treat with, and whether the treatments are working.

The distinction between disease status and activity is critical as demonstrated by experience in the osteoporosis field. In the osteoporosis field, Bone Mineral Density (BMD) provides a biomarker of disease status reflecting the current amount of bone. In contrast, biochemical markers of bone formation (e.g., osteocalcin, PINP), and bone degradation (e.g., CTX-I, NTX-I), reflecting bone cell activity, hence disease activity, provide indications of the underlying pathology preceding changes in disease status. Both BMD and soluble biochemical biomarkers independently predict fracture [6], albeit only one of these (biochemical biomarkers) is an activity measure. In fact, all bone treatments change the bone biochemical biomarkers prior to an effect on BMD, and only treatments that affect the bone biomarkers have been shown to have an effect on BMD [7], and eventually fractures. These concepts are illustrated in Fig. 1. In direct concordance, in the FNIH OA study, the AUC of disease activity biomarkers reflecting either more degradation or less formation of cartilage, were predictive of cartilage loss [8].

**Fig. 1:**
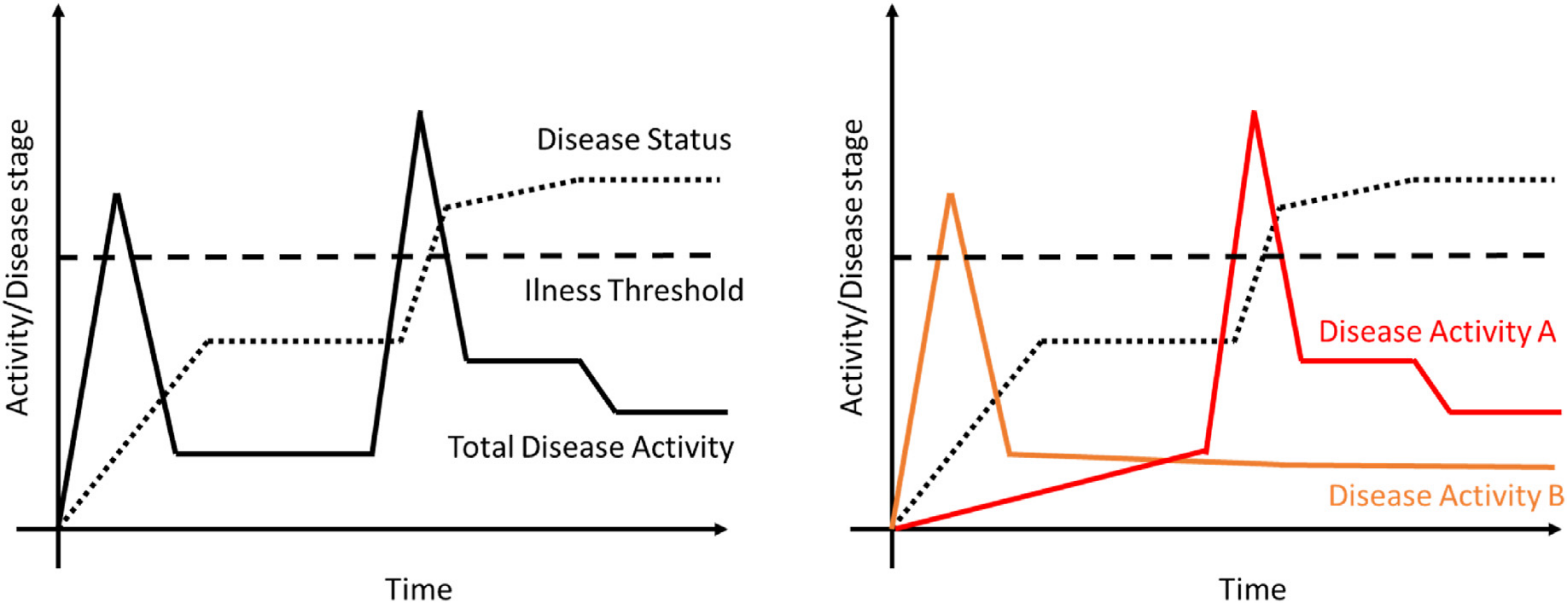
Schematic illustration of disease activity versus disease status. A: Total disease activity may be independent of disease status. OA in particular has been demonstrated to have a dis-concordance between disease activity and status with long periods of inertia followed by fast progression [9]. We begin to appreciate that OA has multiple drivers of disease, most likely at different stages of disease [10,11], as illustrated in Fig. 1. The actual total disease activity may be divided into different types of disease activity at different stages of the disease trajectory. The total disease activity drives the pathology past the illness threshold, resulting in considerable pain and loss of function leading the individual to see a doctor, and likely yielding the diagnosis of a symptomatic OA patient. However, if we target a treatment to the disease activity driver A with a potential treatment that may work against disease driver B, there may be no benefit to the patient. This, treating the symptoms, i.e. the illness (pain), may not correct the underlying diseases. This may in part explain some of the discordance between structure and symptoms in OA.

Our main concern is the underappreciated issue that disease activity in OA may take many forms, although all forms lead to symptomatic disease and joint failure [10,12,13]. A specific treatment targeted to a specific disease activity may be efficacious only for that disease activity. If we hope to be treating the underlying disease drivers leading to changes in structure as measured on imaging, and development of pain and dysfunction, we should be targeting disease activity rather than changes in imaging parameters/readouts. Of note, in some aspects disease activity may be monitored by these tools for better and more sensitive detection of pharmacodynamic changes.

We wish to highlight three issues that warrant further consideration:

1. **Pain is an indicator of illness, not disease status.** Generally, there is only a weak association between pain and disease status, as pain system undergoes plastic changes in association with pain intensity and duration [14]. Unfortunately, pain perception and causes are highly heterogeneous and can be confounded by pain from other non-OA etiologies. To develop and approve a DMOAD we need to better understand the disease. Notably, a reduction of total joint replacement (TJR) numbers/rates alone does not constitute a DMOAD. Consider for example the possibility that a strong pain reliever (an ‘illness modifier’) could postpone TJR, although the drug mode of action may not be on the critical path of disease pathogenesis and, not a ‘disease activity modifier’, consequently resulting in more TJRs with overall less pain [[15], [16], [17]] in the overall population, as seen with the nerve growth factor inhibitor programs. True DMOADS should have mechanisms of action (MOA) on the critical path as described by the FDA in the 2018 revised draft guidelines for drug development of OA [18] and therefore be disease activity modifying OA drugs (DAMOADs), also with symptom/illness modifying effects. To refine treatments for pain modification alone, which is a right on its own, ICD11 codes are now available for primary and secondary pain. Similarly, mechanism-based pain stratification of OA patients have been suggested as a way to ensure that the right patients are recruited for a specific trial [19] from among patients with painful knee OA [20].
2. **Disease activity versus disease status and targeting of the patient population.** Some soluble biomarkers tell us about the disease processes, tissue formation and tissue degradation. As there are numerous tissues involved, as well as catabolic and anabolic processes, we anticipate that combinatorial soluble biomarkers will likely be the most robust indicators of the disease activity in the joint organ. Consequently, treatments should be directed to the elevated level and type of disease activity, i.e. targeting bone, cartilage, tendons, muscle, synovium or another pivotal driver. Only patients with levels of the target disease activity biomarkers, indicating the specific driver of disease, should be targeted and expected to experience a beneficial effect by that treatment.
3. **One size does not fit all MOA development and outcome paths** [21]. All MOA may not provide the same relation between joint structure protection and pain relief. Whereas some interventions may yield concordant outcomes on pain and structure, others may be discordant. For example, a pro-anabolic, i.e., a driver of cartilage formation, may not reduce the main drivers of pain and synovitis. The FDA and EMA are unlikely to approve DMOADs that affect disease activity but do not reduce pain. Rather, it may increase pain by supplying more “fuel” for the damage associated molecular patterns (DAMP) ‘fire’. As the growth factor, FGF-18, induces cartilage formation, at the same time causes increased proteoglycan turnover. This proteoglycan turnover is driven by ADAMTS activity, resulting in the release of the aggrecanase cleaved fragment of aggrecan, known as the ARGS epitope; such released epitopes have been shown to be DAMPs [22], and to drive toll like receptor (TLR) activation and thereby eventually also pain [23]. In caution, while a given treatment may be targeted to a given specific endotype [24], such as a high bone resorption endotype, a MOA exclusively targeting bone may not suffice for both pain and structure, leafing to better outcomes. In further alignment, different endotypes described by selected important biomarkers, may be used to identify more treatable endotyoes [24].

Are we setting ourselves up for further catastrophic failures with the current approaches and outcome measures? If pain and structure are not concordant, then the only solution for MOA on the joint critical path may be outcome studies? We suggest that pain relief may not be the optimal solution for patients, unless that pain modification is concurrent with a modulation of the underlying disease activity. This may suggest new types of outcome studies, i.e. disease activity-based outcome studies. These outcome trials may be enriched for the right disease activity to match the tested pharmaceutical agent and MOA. This may be a refinement, but is in direct alignment with the recent FDA communication on enrichment strategies for outcome studies in OA [25], which was presented by key authors at the CTS. Upon reflection, as Illness represents symptoms of pain and dysfunction with which the patient presents to the doctor's office” – disease activity status and illness are therefore inextricably entwined, regardless of potential discordance of the temporal relationship between them. This suggests that illness must be part of the suite of outcomes tested in a clinical trial. Therefore, this may be an argument for a conditional approval type pathway for a drug and needs to be explored further.

Is this an un-circumventable hindrance for drug development in the OA field, both for phase III study design and investments? We believe not. We believe it is possible to draw inspiration from the osteoporosis field [7], which has event rates for fractures comparable to TJRs in OA (below 1%/year). Several drugs for osteoporosis have been approved in reasonably sized studies; Abaloparatide [26] is an example of a recently approved osteoporosis drug based on a phase III study of 2500 patients followed for 18 months. Osteoporosis phase III studies are enriched by the FRAX [27] algorithm, and refined disease activity parameters, which predict fractures. BMD has been accepted/validated as a surrogate marker in OP that is predictive of outcomes; unfortunately, we do not have a comparable scenario in OA yet. This intermediary step may need to be crossed before we can have successful DMOAD phase III trials. Possibly, it is time that we enrich OA studies for the optimal disease activity parameters that fit the MOA of the intervention in order to generate the needed positive phase III clinical studies in OA for the benefit of patients. This philosophy is in alignment with the recent conclusions from the FDA guidance entitled, “Concept endpoints informing design considerations for confirmatory clinical trials in OA” [25].

## Declaration of competing interest

The authors declared no conflicts of interest.
